# Heterogeneity and emergent behaviour in the vascular endothelium

**DOI:** 10.1016/j.coph.2019.03.008

**Published:** 2019-04

**Authors:** John G McCarron, Calum Wilson, Helen R Heathcote, Xun Zhang, Charlotte Buckley, Matthew D Lee

**Affiliations:** Strathclyde Institute of Pharmacy and Biomedical Sciences, University of Strathclyde, 161 Cathedral Street, Glasgow G4 0RE, UK

## Abstract

The endothelium is the single layer of cells lining all blood vessels, and it is a remarkable cardiovascular control centre. Each endothelial cell has only a small number (on average six) of interconnected neighbours. Yet this arrangement produces a large repertoire of behaviours, capable of controlling numerous cardiovascular functions in a flexible and dynamic way. The endothelium regulates the delivery of nutrients and removal of waste by regulating blood flow and vascular permeability. The endothelium regulates blood clotting, responses to infection and inflammation, the formation of new blood vessels, and remodelling of the blood vessel wall. To carry out these roles, the endothelium autonomously interprets a complex environment crammed with signals from hormones, neurotransmitters, pericytes, smooth muscle cells, various blood cells, viral or bacterial infection and proinflammatory cytokines. It is generally assumed that the endothelium responds to these instructions with coordinated responses in a homogeneous population of endothelial cells. Here, we highlight evidence that shows that neighbouring endothelial cells are highly heterogeneous and display different sensitivities to various activators. Cells with various sensitivities process different extracellular signals into distinct streams of information in parallel, like a vast switchboard. Communication occurs among cells and new ‘emergent’ signals are generated that are non-linear composites of the inputs. Emergent signals cannot be predicted or deduced from the properties of individual cells. Heterogeneity and emergent behaviour bestow capabilities on the endothelial collective that far exceed those of individual cells. The implications of heterogeneity and emergent behaviour for understanding vascular disease and drug discovery are discussed.

**Current Opinion in Pharmacology** 2019, **45**:23–32

This review comes from a themed issue on **Cardiovascular and renal**

Edited by **Frances Plane** and **Paul M Kerr**

For a complete overview see the Issue and the Editorial

Available online 18th April 2019

**https://doi.org/10.1016/j.coph.2019.03.008**

1471-4892/© 2019 The Authors. Published by Elsevier Ltd. This is an open access article under the CC BY license (http://creativecommons.org/licenses/by/4.0/).

;1;

## Introduction

The endothelium regulates virtually every cardiovascular activity by releasing numerous vasoactive agents. To regulate blood flow and blood pressure, endothelium-derived nitric oxide and prostacyclins promote vasodilation, whereas production of endothelin, superoxide and thromboxane stimulate vasoconstriction [1]. Blood fluidity is regulated by endothelial factors that interrupt the blood clotting cascade (antithrombin III, the protein C receptor thrombomodulin, and tissue factor pathway inhibitor [2]), platelet activation (nitric oxide and prostacyclin, exonucleotidases and surface heparan sulphates [2]) and fibrinolysis (tissue-type plasminogen activator and its inhibitor plasminogen activator inhibitor-1 [3]). Angiogenesis is regulated by the endothelium receiving signals from molecules such as vascular endothelial growth factor (VEGF) and von Willebrand factor [4, 5, 6, 7, 8]. Blood vessel permeability plays a key role in tissue perfusion and host defence and is regulated by the expression of endothelial cell adhesion molecules. Endothelial cells also play a key role in immune and inflammatory reactions by regulating leukocyte movement into tissues via production of chemokines, colony stimulating factors and expression of specific proteins and cell adhesion molecules at sites requiring defence or repair [9, 10, 11].

In making ‘decisions’ on vasoactive outputs, the endothelium must coordinate responses across many cells to numerous local and circulating cues. The cues are generated by changes in physiological status and contained in signals within the chemical environment to which the endothelium is exposed. The environment is complex. Signals are received in the form of the blood composition (e.g. pH, O_2_), hormones, neurotransmitters, and from pericytes, smooth muscle cells, various blood cells, viral or bacterial infection, proinflammatory cytokines and even endothelial cells themselves. Multiple signals may arrive simultaneously, each providing separate instructions to the vascular system [12, 13, 14, 15, 16]. To manage and accurately process the information, the endothelium must selectively and sensitively resolve each message held within the chemical environment while remaining resistant to the random spontaneous activity in cells (noise).

Even a single message may itself vary widely in intensity and carry different instructions at different concentrations. Major physiological crisis events, such as bleeding, may generate large local concentrations of activators [17] to evoke substantial immediate cardiovascular responses, for example, blood clotting. However, a great deal of cardiovascular activity is routine, small background adjustments driven by modest changes in circulating activators. Fluctuations in the concentration of agents that activate the endothelium to produce background physiological adjustments are vanishingly small and require the endothelium to be exceptionally sensitive. The concentration changes of circulating hormones, such as leptin, estradiol, parathyroid hormone, epinephrine, angiotensin II are small and vary periodically from a basal value of a few tens of picomolar to a peak rise in the low hundreds of picomolar [12, 13, 14, 15,18]. Leptin, for example, varies periodically over a 24 hour period from a basal concentration of ∼49 pM to a peak of 99 pM (8–16 ng/ml) [12]. Estradiol oscillates between 74 pM (20 pg/ml) and 220 pM (60 pg/ml) though occasionally reached a unusually high concentration of 1.5 nM (400 pg/ml) [12]. Parathyroid hormone from 5 pM (50 ng/l) to 80 pM (75 ng/l) [13] and epinephrine from 273 pM (50 pg/ml) to 2.2 nM (400 pg/ml) [19]. Even during a fight-or-flight crisis epinephrine concentration may reach only ∼55 nM (10 ng/ml) [20]. Each of these concentrations would be considered very low by conventional pharmacological standards. Yet the high-sensitivity enables the endothelium to effectively monitor and respond to the circulating activators to maintain ‘background’ cardiovascular activity.

However, a problem coupled to the high-sensitivity of the endothelium is how random ‘noise’ occurring in the detection system can be rejected. Random noise is unavoidable and arises from stochastic processes that occur in the cells themselves. Random noise can evoke signals of a substantial magnitude. The endothelium must recognise these events as noise and prevent them from evoking widespread activity while retaining high-sensitivity. These features of endothelial sensitivity raise a number of questions. How does the endothelium efficiently detect exceptionally low concentrations of activators associated with background physiological events, while also being able to respond to very high concentrations during crisis (e.g. bleeding) events? How is noise distinguished from ‘real’ signal? By what mechanism does the endothelium process multiple, separate, and even contradictory, messages? What underlies the change in endothelial sensitivity that occurs in cardiovascular disease? The answers to these questions are largely unknown but will provide fundamental insight into endothelial function and the dysfunction occurring in cardiovascular disease.

In this review, we outline key properties of the endothelium that are involved in the detection of the numerous signals required for cardiovascular activity. We highlight how the behaviour of individual endothelial cells differs from expectations derived from the most common experimental approaches used to study the endothelium and the impact these differences may have on drug discovery.

## Uniformly heterogenous

Given the differences in haemodynamics, physiology and structure of blood vessels, it is not surprising that endothelial cell specialisation occurs in different parts of the vascular system [21, 22, 23, 24, 25, 26, 27, 28, 29, 30, 31, 32, 33,34^•^]. However, within a region of a blood vessel, most studies examining sensing and activation treat the endothelium as a homogenous population of cells that respond uniformly. Each cell, it is believed, detects each signal equally, and each cell’s response is considered to be a miniature version of the entire response. An attractive feature of the proposal is that no additional consideration is required to explain coordinated function of the endothelium; coordination is achieved by uniform activation of cells.

The hypothesis that endothelial cells behave uniformly is implicit in most experimental approaches used to study the endothelium. In organ bath experiments, the mechanical response of the artery or vein is used as an indirect measure of endothelial activity. This type of experiment has yielded many important insights into endothelial function (e.g. discovery of nitric oxide) but averages the behaviour of thousands of endothelial cells and assumes each cell behaves in a similar way. The organ bath approach is not unique in assuming uniformity. Many current interpretations of gene expression, protein levels or metabolic signalling that are derived from immunoblots, PCR or microarrays also assume that all cells of a population are comparable in receptor complement and signalling processes [35,36]. When changes are reported in cardiovascular disease, it is assumed all cells are altered equally.

**Figure 1:**
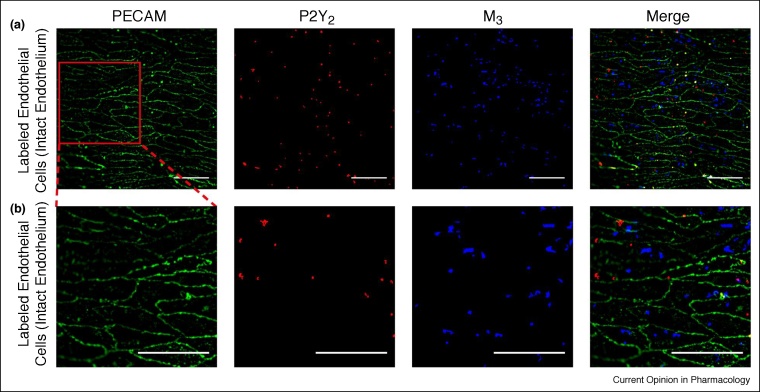
Heterogenous distribution of muscarinic and purinergic receptors. **(a)** Fluorescence localisation of purinergic P2Y2 receptors and muscarinic M3 receptors in the endothelium. Representative images (from left) show the endothelial cell boundaries as revealed by PECAM-1 labelling (green; anti-CD31/PECAM-1), P2Y2 receptor (red; anti-P2Y2) distribution, M3 receptor distribution (blue; fluorescent M3 receptor antagonist, 100 nM) and overlay of all three. The receptors distribution was not uniform across the endothelium and there was relatively little overlap of purinergic and muscarinic receptor staining. **(b)** Expanded view of the endothelial images shown in (a). The expanded region is shown by the red box in (a) left-panel. All scale bars = 50 μm. Modified from Ref. [50^•^] with permission.

While the hypothesis of homogenous cell responses is attractive and widely accepted, the hypothesis has not been confirmed at the level of individual cells. When individual cells in a population have been examined, differences in the time course of responses or expression of proteins have been almost universally reported [37, 38, 39, 40]. For example, signalling in cells occurs out of phase with neighbouring regions and receptors are heterogeneously distributed [21,41, 42, 43, 44, 45, 46, 47]. The distribution of angiotensin II immunostaining is irregular in neighbouring endothelial cells of femoral mesenteric artery [30]. An uneven mosaic pattern of von Willibrand factor (VWF)-positive and von Willibrand factor (VWF)-negative endothelial cells occurs in many vascular beds and even in capillaries [30,48^•^,^49^]. Adrenergic α-adrenoceptor clusters and cannabinoid receptor distribution are heterogeneous among cells in the endothelium [42]. There is also heterogeneity in the distribution of muscarinic (M3) receptors and purinergic (P2Y2) receptors in neighbouring cells (Figure 1; [50^•^]).

Functional data also show heterogeneity in endothelial responses. Acetylcholine-evoked Ca^2+^ responses are larger at branches in rat thoracic aorta than that of neighbouring non-branch regions [51^•^]. The reverse is true of histamine [51^•^]. Studies of murine thoracic aorta endothelial cells that used only single concentrations of agonists found that while most cells (82%) responded to ATP, large fractions of cells did not respond to acetylcholine, bradykinin or substance P [52]. Responses to mechanical activation also are not uniform and certain populations of cells are more likely to respond to shear stress [53].

Our results also demonstrate heterogeneity in the endothelium’s response. Different cells respond to different concentrations of the activator acetylcholine [54^•^]. As the concentration increased, an increasing number of cells were recruited. The increasing recruitment of cells was part of the concentration-dependence of the response. While the sensitivity range of the endothelium as a whole to acetylcholine extended over three orders of concentration magnitude, each was sensitive over only one order of magnitude [54^•^], that is, the system has properties that differ and exceed the properties of individual parts (cells). This arrangement (having different cells sensitive to various concentrations) solves a problem common to sensory systems, that is, how to create a system that is exceptionally sensitive to a stimulus but does not saturate at low-stimulus intensity. The endothelium’s organisation solves this problem by having individual cells that are highly sensitive detectors of very limited concentration ranges [54^•^].

Interestingly, cells that were comparably sensitive were positioned close together in discrete clusters. As the concentration of each agonist increased, more cells in the cluster activated and more clusters responded [50^•^,54^•^]. These findings revealed that sensing cells are neither randomly nor uniformly distributed, but structured into sensing regions [54^•^].

The clustering of cells creates properties of the system that are absent in single cells and generates a mechanism to help reject noise. Clustering may provide a co-incidence detection system [50^•^,54^•^]. When single cells in the endothelium were activated (as would occur in stochastic noise events), there is little propagation of signals occurred to neighbouring cells. However, when two or more neighbouring cells activated together, signal propagation occurred. By rejecting noise, signal detection will improve and randomly occurring events are unlikely to be propagated. These observations again show that the endothelium as a whole has properties that are quite distinct from those of individual cells. Clustering may offer other advantages. Clustering may allow the uptake and breakdown mechanisms for diffusible messengers (e.g. nitric oxide, prostaglandin) to be overwhelmed, providing increased spillover of signals. Clustering may also limit interference from neighbouring cells that are responding to a different stimulus, that is, a single cell responding in isolation may easily be influenced by neighbouring cells and have its signal overridden. A cluster of cells each performing the same task may be much harder to override.

The mechanisms giving rise to the organisation of cells into clusters are not yet clear. Perhaps self-replication occurs during development or the cells may be at different developmental ages because of cellular renewal. Alternatively there may be feedback control of function and receptor expression based on location or a result of a self-organisation process at the cellular level.

## Fractured but whole; communication and cooperation

While the system appears to be fractured and operating as a series of distinct clusters of cells, communication ensures the endothelium functions seamlessly as a harmonised whole. Each endothelial cell is connected to approximately six neighbouring cells (Figure 1; [50^•^]) and interaction occurs among connected cells. Communication via gap junctions [55,56] is an acknowledged feature of the endothelium [55,57, 58, 59], and the low electrical resistance (∼5–70 MΩ [60, 61, 62, 63, 64]) demonstrates the high extent of connectivity among cells.

The communication pathways create a network capable of relaying information [55,57, 58, 59,65]. However, the communication pathways in the endothelium are usually treated as being like a wire in a telecommunication system. An input to the system is relayed from cell to cell but decays passively with distance, as described by the systems ‘cable properties’ [66]. The network itself is not thought to interact with the input but relays signals without changing the signals characteristics. In other systems with similar passive network properties, separate input signals may passively summate or cancel in an approximately linear way. In this regard, the system has linear features with ‘resultant’ properties which can be predicted. The resultant may be a sum or a difference of the interacting elements.

**Figure 2:**
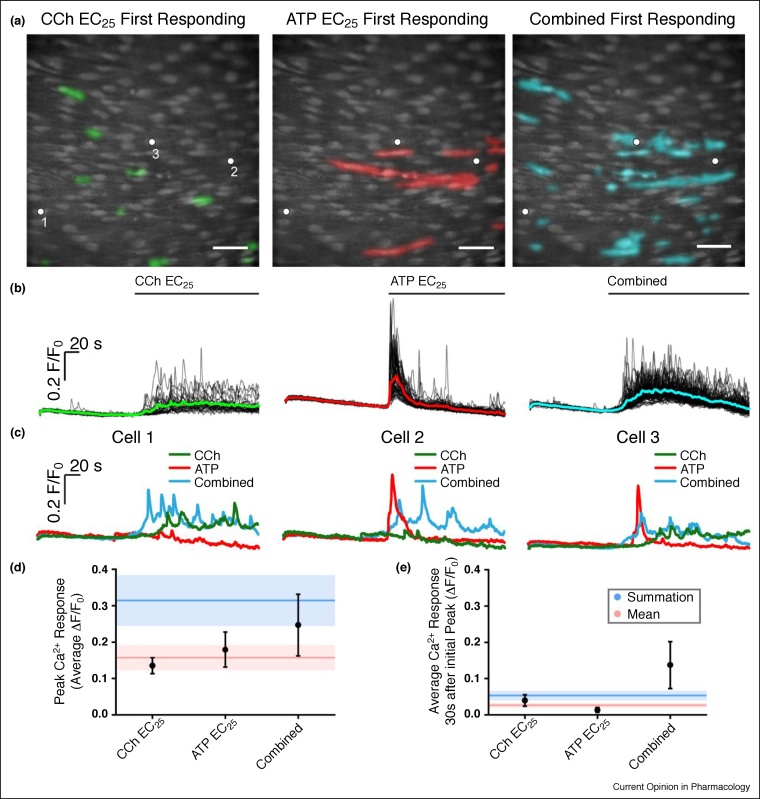
Unique signals in separate cells. **(a)** Composite Ca^2+^ images showing cells that respond in the first 4 s of activation by the EC_25_ concentrations of CCh (left, green) and ATP (middle, red). Images are of the same field of endothelium. The right-panel shows cells activated (cyan) in the same field of endothelium when both drugs were applied together. **(b)** Ca^2+^ responses from all activate cells in the field of endothelium shown in (a). The Ca^2+^ increase evoked by CCh was (on average) a slow increase that remained elevated on which repeating oscillations occured (left green). The response to ATP on average was a sharp transient increase that declined towards resting values (red middle). When both agonists were applied (combined; blue right) the Ca^2+^ increase appeared to have features of each agonist, that is, a slow but larger initial increase than with CCh, which remained more elevated than ATP and slowly declined. Agonists were present for the duration indicated by the line above each trace. **(c)** Examples of responses from three separate cells to CCh, ATP and to the two agonists when applied together. In each panel in (c), traces are from the cells indicated by the white dots in the panels in (a). It is the same three cells in each case. Cell 1 is shown in the left panel in (c), cell 2 in the middle panel and cell 3 in the right panel. Cell 1 (left panel) responds to CCh but not to ATP. The characteristics of the response in Cell 1 is altered when both ATP and CCh are present (combined) with a faster and larger upstroke. Cell 2 responds to ATP but not CCh. Once again, the characteristics of the response in Cell 2 is altered when both ATP and CCh are present (combined) with a more sustained later Ca^2+^ change. Cell 3 responds to each agonist (CCh and to ATP). Once again, the characteristics of the response in Cell 3 is altered when both ATP and CCh are present (combined). **(d)** Mean peak responses (black circles) to the EC_25_ of CCh and ATP separately and when both were present together (combined). The red line shows the calculated mean of peak response when both agonists were added separately. The red shaded region shows the standard error of the mean. The blue line shows the sum of the peak responses when both agonists were added separately. The blue shaded region shows the standard error of the mean. The combined peak response exceeded the mean and was less than the summed response. **(e)** Mean steady-state responses (black circles) to the EC_25_ of CCh and ATP separately and when both were present together (combined). The red line shows the calculated mean of the steady-state response when both agonists were added separately. The red shaded region shows the standard error of the mean. The blue line shows the sum of the steady-state responses when both agonists were added separately. The blue shaded region shows the standard error of the mean. The combined steady-state response exceeded both the mean and the summed response. All Scale bars = 50 μm. From Ref. [50^•^] with permission.

However, in contrast to the simple resultant properties of a system, an incredible feature of the endothelium is that the system may interact with the inputs to generate new distinctive signals that differ in a non-linear way from the inputs. The muscarinic agonist carbachol and purinergic agonist ATP, activate spatially separate clusters of cells [50^•^]. In those separate clusters of cells, Ca^2+^ signals evoked by carbachol and ATP are distinctive [50^•^]. Carbachol evokes oscillating Ca^2+^ signals, ATP evokes an initial Ca^2+^ spike followed by a decline towards basal values (Figure 2). This observation demonstrates that an input/output relationship exists for each agonist that is temporally as well as spatially distinctive, that is, the endothelium can distinguish inputs and assign outputs (Figure 2).

While most endothelial cells are heterogeneous and particularly sensitive to one activator, a small number of cells are sensitive to more than one activator [50^•^]. When the agonists are added separately, these cells also generated distinctive signals to each agonist and the signals were similar to those seen in cells sensitive to only one activator (Figure 2; [50^•^]). This observation suggests that the distinctive signals evoked by each agonist are a feature of the agonist acting on the cells rather than the cell itself.

*Emergent signals.* Surprisingly, even though separate cells are activated by muscarinic and purinergic agonists, when carbachol and ATP were present together, the resulting Ca^2+^ signal is distinct from those signals generated by either of the agonists in isolation (Figure 2; [50^•^]).

The change in the response occurring, when both agonists were present, is particularly interesting. The change in the steady-state response is expansive in that it far exceeds either the average or summation of the two inputs (Figure 2; [50^•^]). The generation of a distinct new signal suggests that cells perform signalling computations and combine information from multiple sources. The change in signal characteristics occur even though distinct, separate cells were activated by each agonist, that is, communication occurs between the separate clusters (Figure 2; [50^•^]). The computations on the signals show the endothelium is flexible and interacts with the inputs to generate new signals.

The precise nature of the computation carried out on the signals and the underlying mechanisms are not clear. Nonetheless, the computation is important in that it is a feature that emerges from the collective dynamics of the endothelial network and provides a mechanism for the endothelium to interactively monitor external environments via distributed sensing across separate cells. Examining the endothelial system as a whole reveals properties that are greater than the sum of its parts.

## Emergent properties: why organise with heterogeneity?

**Figure 3:**
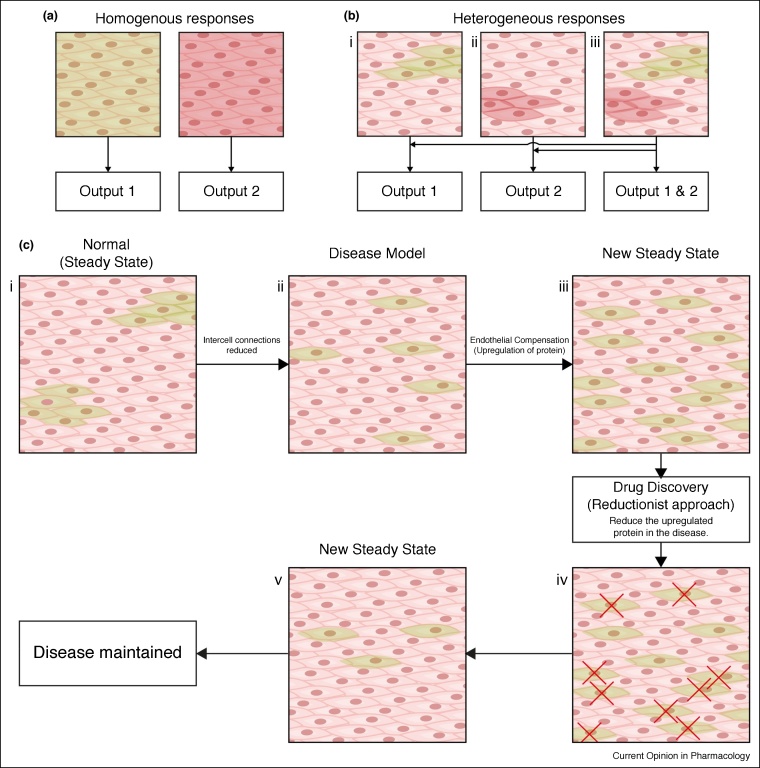
Uniformity, heterogeneity and reductionism. **(a)** Illustration of a homogenous population of cells responding to single stimuli. Each cell responds uniformly to either activator (green or red) and generates one output to each stimulus. **(b)** Heterogeneous populations of cells responding to one (bi and ii) and two (biii) different stimuli. Separate spatially distinct clusters of cells process and respond to each activator to generate specific outputs. When both activators are present together (biii) the separate regions respond and multiple outputs can be generated. This arrangement permits the endothelium to simultaneously process different stimuli in parallel. **(c)** Illustration showing a reductionist approach to drug discovery. The normal, steady-state behaviour present in health (ci) may be disrupted in disease (cii). The endothelium may compensate for this alteration by upregulating proteins in other cells to restore a near normal steady-state in disease (ciii). A reductionist approach to drug discovery, that measures the individual components, may attribute this upregulation to the dysfunction in disease rather than the compensatory mechanism employed by the endothelium to overcome the disease. Targeting this upregulated protein may force the endothelium into another new steady-state that is not beneficial (civ) which lacks the compensatory mechanism (cv) present before pharmacological intervention.

The interactive collective activity of the cells may hold the key to resolving some of the incredible virtues of the endothelium (Figure 3). Endothelial heterogeneity permits different cells to acquire, in an exceptionally sensitive manner, different elements of the overall information available. However, while cells detect a very limited aspect of the total information content, information is shared so that, collectively, the endothelium interprets the entire chemical environment. As a collective, the endothelium has properties that exceed the capabilities of single cells. This feature is not unique to the endothelium. Biological systems are recognised increasingly as having properties that are distinct from the individual components of the system. New distinct features often arise from interactions and give rise to behaviours that are absent when cells are examined in isolation.

New features that appear in a system and which differ from the expectations of the sum of the components are called ‘emergent properties’ [67,68]. Emergent properties differ significantly from those observed in linear (resultant) systems. Unlike linear systems where the whole is equal to the sum of the parts, emergent systems arise from non-linear interactions and create new collective behaviours that make the whole much greater than the sum of the parts. Emergent properties cannot be reduced to properties of the constituent parts of the system and resist attempts at being inferred or predicted by calculation. In the case of the endothelium, distinctive signals are generated to different agonists, and signals appear in the system that are quite different from the sum of the inputs when multiple agonists are present [50^•^,54^•^]. The difference between the behaviour of individual cells and the population average result in the endothelium as a whole being capable of processing more information, more precisely, than cells acting alone. These features have important implications for basic investigations on endothelial function and on drug discovery.

*Emergent behaviour in cardiovascular disease.* The underlying rationale of most basic investigations is the desire to understand the components that give rise to a response. In disease, these components identify targetable processes, for example, protein kinases, receptors or ion channels, for small molecule design. Most drug discovery investigations rely on averages from population (cells or tissue) studies. However, this reliance assumes uniform behaviour in populations, and believes an understanding of how each cell works will results in an understanding of the system, that is, the investigations are driven by reductionism.

In the reductionist approach, a larger system is analysed by breaking it down into pieces and determining the connections between the parts [69]. Isolated cells, proteins, molecules and ions have sufficient explanatory power to provide an understanding of the whole system and the changes that occur in disease (Figure 3). Reductionism has provided a wealth of knowledge on cellular components and their function. However, despite the success of reductionism, it is increasingly clear that biological function can only rarely be attributed to isolated components.

Reductionist approaches have generated insights into how cells work within a system; however, drug discovery requires an understanding of how the overall system works [70]. Yet, drug discovery is largely based on reductionist approaches such as genomics, proteomics, metabolomics, high-throughput screening, combinatorial chemistry and bioinformatics [70]. These approaches have not brought the new products that were anticipated [71,72]. For example, knowledge of the genome sequences of humans and various pathogenic agents, once hailed as the step change opening doors to new drug development, and ‘personalised’ or ‘precision’ medicine, has led to the identification of only a limited number of beneficial drug targets [73]. Gene therapy, stem-cell research, antisense technology and cancer vaccines have not materialised to the degree so feverously predicted by early supporters [72]. Despite eye-watering financial investment, sixty years of reductionist approaches involving molecular targeting of drugs for specific enzymes and receptors in cancer chemotherapies, have failure rates of 90% in those agents managing to reach end-stage trials [74^•^].

For cardiovascular drug discovery, an understanding of the behaviour of the endothelium requires an appreciation of the multiple non-linear interactions and feedbacks that occur within and among cells. The interactions and feedbacks are currently poorly understood. Unfortunately, even if a near complete understanding existed, the behaviour of the system would remain difficult to predict and reconstruct. Altering the function of a component in the system will have rippling unintentional consequences, because of feedbacks and interactions, that may be difficult to foresee and may rarely be beneficial (Figure 3). Key to understanding the endothelium is one defining principal – the endothelium exists in a complex steady-state. The system resists changes and will continuously work back (asymptotically) towards the steady-state value.

In a cardiovascular disease, alterations of key components (enzymes, ion channels) may trigger a change which forces the entire system into a new steady-state, albeit one that is dysfunctional. However, the new dysfunctional condition will once again be maintained in a steady-state by multiple altered interactions and feedbacks occurring among enzymes, metabolic processes, ion channels and so on. As a result, there are multiple changes in the function of the components of the system (e.g. enzymes, ion channels). Most of the changes will be consequences rather than causes of cardiovascular disease and are, in fact, beneficial acting to stabilise the system and halt or limit the progression of the disease (Figure 3). The triggering, initiating, event may not be easy to identify among the changes. Therefore, targeting pathways blindly, as is done through reductionism (e.g. identified with proteomics), may have adverse effects as stabilising ‘beneficial’ changes will almost certainly be targeted. It may be fruitless to attach any particular significance to changes in specific biomarkers/proteins (e.g. ion channels, enzymes) in cardiovascular disease — they may be consequences rather than causes of the disease. Pharmacologically altering the behaviour of a biomarker/protein may not restore the system. Instead, these pharmacological agents may cause additional changes in overall function of the system and yet another new steady-state could arise. The interactions and feedbacks that occur in complex systems may explain why the development of many, perhaps most, successful clinically used drugs has been through serendipity rather than rational drug design [75]. A successful approach for rational therapeutic development in any system with emergent properties will require an understanding of the multiple interactions among vital components that support the entire network’s structure and function, and how the interactions change in cardiovascular disease.

## Conflict of interest statement

Nothing declared.

## References and recommended reading

Papers of particular interest, published within the period of review, have been highlighted as:

• of special interest
